# Association between abdominal obesity and cognitive outcomes in middle-aged adults: a systematic review

**DOI:** 10.3389/fnut.2026.1879986

**Published:** 2026-09-08

**Authors:** Lichao Wang, Yang Cao, Zixu Wang, Huazhong Xiong, Yunlong Xu, Jixiang Ren

**Affiliations:** 1 College of Traditional Chinese Medicine, Changchun University of Chinese Medicine, Changchun, China; 2 Affiliated Hospital of Changchun University of Chinese Medicine, Changchun, China

**Keywords:** abdominal obesity, central obesity, cognitive decline, cognitive outcomes, middle-aged adults, systematic review

## Abstract

**Objective:**

This systematic review aimed to evaluate the association between abdominal obesity and cognitive outcomes in middle-aged adults and to summarize the underlying biological mechanisms and potential modifying factors, with intervention evidence discussed as a complementary clinical perspective.

**Methods:**

We systematically retrieved relevant literature published from January 2000 to May 2026 across PubMed, Embase, Web of Science, Cochrane Library and CNKI databases. Eligible observational and interventional studies focusing on middle-aged adults were included. Two independent researchers strictly completed study selection, data extraction and methodological quality assessment in accordance with the PRISMA 2020 reporting guidelines.

**Results:**

A total of 96 qualified studies were finally included for systematic analysis. Abdominal obesity assessed by waist circumference, waist-to-hip ratio and other central adiposity indicators was consistently associated with poor cognitive performance and elevated risk of cognitive decline in middle-aged populations. Multiple biological mechanisms were involved, including chronic inflammation, oxidative stress, insulin resistance, neuroendocrine dysregulation, mitochondrial dysfunction and gut-brain axis imbalance. Dietary patterns, physical activity and sleep quality effectively modify this association, while genetic susceptibility and long-term chronic stress further increase cognitive impairment vulnerability. Lifestyle interventions exert positive effects on improving metabolic status and cognitive function in short-term trials, whereas high-quality evidence for pharmacological treatment, surgical intervention and traditional Chinese medicine remains relatively limited and requires further investigation.

**Conclusion:**

Current evidence supports a clinically relevant association between greater midlife central adiposity and poorer cognitive outcomes across a spectrum from domain-specific deficits to cognitive decline and dementia-related endpoints. The evidence is biologically coherent but does not establish that abdominal obesity is independently causal in every population or that reducing waist circumference alone will prevent cognitive decline. Future studies should standardize abdominal obesity severity categories and cognitive impairment grades, use repeated exposure and cognitive measurements, and integrate neuroimaging and biomarker data to clarify dose-response, mediation, and temporality.

## 1 Introduction

Abdominal obesity (central obesity) in middle-aged adults (45–59 years) is defined by waist circumference (WC) ≥90 cm in men or ≥85 cm in women, typically assessed using WC or waist-to-hip ratio (WHR), with specific criteria following the standards established in the 2024 edition of the Chinese Guidelines for the Diagnosis and Treatment of Obesity (1). With the global prevalence of obesity rising continuously, the association between midlife abdominal obesity and cognitive impairment has become a research focus. Existing studies confirm that abdominal obesity not only accelerates cognitive decline but also increases the risk of neurodegenerative diseases such as Alzheimer's disease (AD) (2–5). Notably, abdominal obesity differs from general obesity in that it primarily reflects visceral adiposity, which is more strongly linked to metabolic dysfunction, chronic low-grade inflammation, and downstream brain-related risks (6, 7).

Cognitive health declines progressively with age, and early metabolic disturbances in midlife have been increasingly recognized as important predictors of later cognitive impairment (8, 9). Midlife represents a critical and potentially reversible window for preventive intervention, during which metabolic dysfunction may still be corrected before irreversible neuropathological changes occur (10–12). Despite these observations, comprehensive systematic reviews focusing specifically on middle-aged populations remain scarce. The underlying mechanisms linking abdominal obesity to cognitive decline involve multiple biological. This review aims to provide a theoretical foundation for future intervention strategies, with the goal of delaying cognitive decline, preventing neurodegenerative and cardiovascular diseases, and informing targeted public health policies.

Complementary approaches, including perspectives from Traditional Chinese medicine (TCM), have been proposed as potential strategies to address cognitive decline associated with abdominal obesity. However, current evidence remains preliminary and exploratory, and further large-scale, high-quality randomized controlled trials are needed to confirm any cognitive benefits (13–15). These intervention findings are discussed as a complementary component of this review, providing clinical context for the association and mechanism findings.

Despite increasing evidence linking obesity to cognitive dysfunction, previous reviews have largely focused on general obesity, older populations, or dementia-related outcomes, whereas the specific role of abdominal obesity during midlife remains insufficiently synthesized. Midlife represents a critical and potentially reversible stage during which metabolic dysfunction may begin to exert adverse effects on brain health before irreversible neurodegenerative changes occur. In addition, abdominal obesity may more accurately reflect visceral adiposity and metabolic risk than general obesity indices such as body mass index (BMI). To our knowledge, no previous systematic review has specifically focused on the association between abdominal obesity and cognitive outcomes in middle-aged adults. Therefore, this review systematically synthesizes epidemiological evidence, candidate biological mechanisms, and modifying factors related to abdominal obesity and cognitive outcomes in middle-aged adults. Intervention implications are included as a supplementary clinical context rather than a primary analytical focus. The aim is to clarify current evidence, identify research gaps, and inform future prevention strategies.

## 2 Methods

### 2.1 Study design and reporting standard

This systematic review was conducted and reported following the Preferred Reporting Items for Systematic Reviews and Meta-Analyses (PRISMA) 2020 statement. The review was not prospectively registered.

### 2.2 Search strategy

We systematically searched PubMed, Embase, Web of Science, Cochrane Library, and CNKI for studies published between January 2000 and May 2026. Search terms included “abdominal obesity,” “central obesity,” “cognitive function,” “cognitive decline,” and “middle-aged adults” (MeSH terms and free words in Chinese and English). Detailed search strategies for each database are provided in Supplementary Table S1.

### 2.3 Eligibility criteria

Studies were included if they met the following criteria: 1. focused on middle-aged adults (defined as 45–59 years, with allowances for studies whose age range substantially overlapped with this definition, provided that data for the middle-aged subgroup could be extracted); 2. examined the association between abdominal obesity (assessed by WC, WHR, WHtR, or visceral fat area) and cognitive outcomes (including global cognition, memory, executive function, attention, cognitive decline, MCI, or dementia); 3. were original research articles, including cross-sectional studies, cohort studies, randomized controlled trials (RCTs), and Mendelian randomization studies. Studies reporting body mass index or general obesity without an extractable central adiposity measure were not used to support abdominal-obesity-specific severity comparisons, although they could be retained as contextual evidence when they also informed the broader obesity-cognition relationship. Review articles, case reports, conference abstracts, and studies with incomplete data were excluded.

### 2.4 Study selection

**Figure 1:**
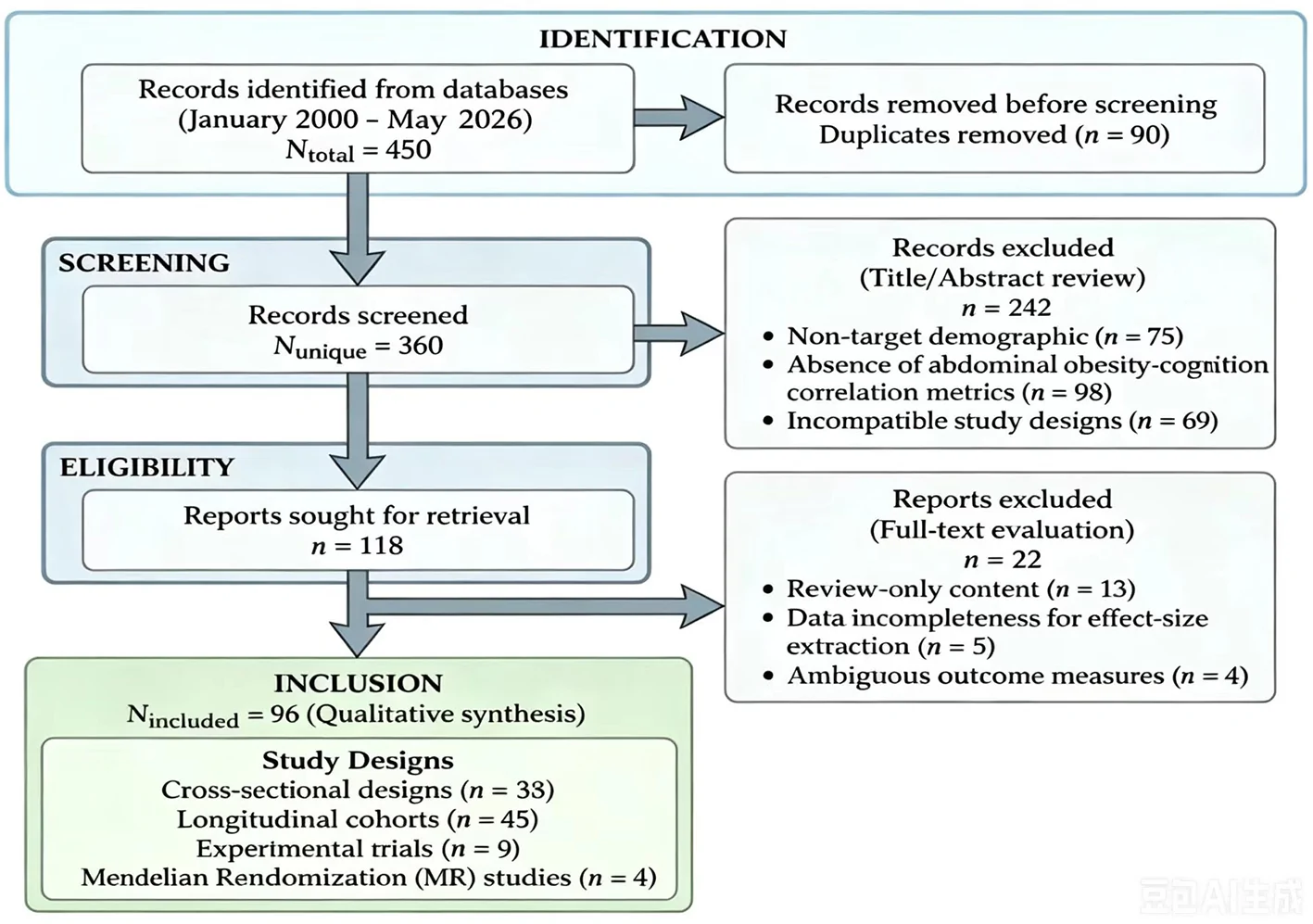
PRISMA 2020 flow diagram for literature screening.

A total of 450 records were retrieved; 90 duplicates were removed; 242 records were excluded after title/abstract screening (non-target population, no core association, or incompatible study designs); 22 were excluded after full-text assessment (13 reviews, 5 with incomplete data, 4 with ambiguous outcome measures), yielding 96 eligible studies. The PRISMA 2020 flow diagram (Figure 1) summarizes the literature screening process.

### 2.5 Data extraction

Data extraction was performed independently by two reviewers using a standardized data extraction form. Extracted variables included study design, population characteristics, abdominal adiposity indicators and cut-offs, cognitive outcome measures, adjusted effect estimates where available, covariates, and study quality indicators. Disagreements between reviewers were resolved through discussion and consensus, and unresolved discrepancies were adjudicated by a third reviewer.

### 2.6 Quality assessment

The methodological quality of included studies was assessed using appropriate tools according to study design: the Newcastle-Ottawa Scale (NOS) for cohort and case-control studies, the Joanna Briggs Institute (JBI) Critical Appraisal Checklist for cross-sectional studies, and the Cochrane Risk of Bias Tool 2.0 (ROB 2.0) for RCTs. Previous systematic reviews were not formally included as primary evidence but were consulted for background and reference tracing.

### 2.7 Data synthesis

Given the anticipated heterogeneity in study designs, exposure measures, and cognitive outcome assessments, the evidence was synthesized narratively. Findings were organized according to study design (cross-sectional, longitudinal, interventional) and thematic categories (epidemiological evidence, biological mechanisms, modifying factors, intervention implications). A quantitative meta-analysis was not performed because of substantial heterogeneity across studies in exposure definitions, cognitive outcome assessments, study populations, and analytical methods. A stratified narrative comparison was additionally conducted using the original study-level effect estimates reported in the included publications, comparing effect direction and magnitude across adiposity severity or exposure contrasts and across cognitive outcome grades. Therefore, a narrative synthesis approach was considered more appropriate.

## 3 Characteristics of included studies

### 3.1 Study design and geographic distribution

A total of 96 eligible studies were included in this systematic review. The included studies comprised cross-sectional studies, longitudinal cohort studies, RCTs, and Mendelian randomization studies. Geographically, the studies were distributed across Asia, Europe, North America, and Africa, with a substantial proportion originating from Chinese population-based cohorts, including the China Health and Retirement Longitudinal Study (CHARLS).

### 3.2 Measures of abdominal obesity

Abdominal obesity was assessed using various anthropometric indicators across the included studies. The most commonly employed measures were WC, WHR, and WHtR. Some studies also utilized the visceral adiposity index (VAI) or imaging-based quantification of visceral fat area. For the purposes of this review, studies were considered eligible if they employed any validated measure of central adiposity.

### 3.3 Cognitive outcomes assessed

Cognitive outcomes assessed in the included studies encompassed global cognitive function [typically measured by the Mini-Mental State Examination (MMSE) or Montreal Cognitive Assessment (MoCA)], domain-specific cognitive functions including memory, executive function, and attention, as well as clinically defined outcomes such as cognitive decline, MCI, and dementia.

### 3.4 Quality of included studies

Quality assessment revealed variability across the included studies. Longitudinal cohort studies generally demonstrated higher methodological quality with clear exposure assessment, adequate follow-up duration, and appropriate adjustment for confounders. Cross-sectional studies provided foundational evidence but were limited by their inability to establish temporal relationships. RCTs varied in sample size and intervention duration, with some exhibiting risk of bias related to blinding and attrition.

### 3.5 Availability of data for stratified association analysis

Not all 96 included studies could contribute to the severity- and grade-stratified comparison, because reporting of stratified estimates was uneven: some studies modeled adiposity continuously, others used categorical thresholds, and longitudinal studies varied in follow-up duration and outcome definitions. Therefore, only studies providing extractable study-level contrasts were included in the stratified analysis.

## 4 Epidemiological evidence

Obesity is a global health crisis. A Lancet study reported that by 2021, China had 402 million overweight and obese adults aged ≥25 years, ranking first globally (16). National surveillance data indicate that WC begins to rise after age 30, peaking after age 50 (17), and that the overall prevalence of central obesity among Chinese adults is 29.9% (18).

Recent work has identified a middle-aged-specific subpopulation of fat precursor cells, CP-A (committed pre-adipocytes, age-enriched), that drives visceral fat expansion during middle age (19). Lineage tracing in mice demonstrates that adipose progenitor cells (APCs) in visceral fat undergo extensive adipogenesis during middle age. Middle-aged mouse APCs possess high cell-autonomous lipid production capacity, while CP-As exhibit elevated proliferation and lipogenic activity. Leukemia suppressor factor receptor signaling is crucial for CP-A lipogenesis and visceral fat expansion. Crucially, identical CP-A cells have been identified in adipose tissue of middle-aged human males, with conserved regulatory signaling pathways, suggesting this mechanism may contribute to the development of abdominal obesity in middle-aged humans (particularly males). This provides a new explanation for the age-dependent characteristics of middle-aged abdominal obesity (19).

Multiple large cohort studies link midlife abdominal obesity to cognitive decline. In Asian populations, visceral fat accumulation correlates with accelerated cognitive aging: each 0.27 kg increase in visceral fat corresponds to 0.7 years of cognitive aging (20). The Northern Manhattan Study confirmed that WHR correlates with processing speed and executive function in middle-aged adults (8). Longitudinal data from the CHARLS cohort similarly show a significant association between abdominal obesity and cognitive decline in Chinese adults (21). A meta-analysis of 5,060,687 participants quantified this risk: each one-standard-deviation (1-SD) increase in WC was associated with a 1.43-fold higher risk of cognitive impairment, 1.53-fold higher risk of dementia, and 1.39-fold higher risk of Alzheimer's disease (AD) (4).

Furthermore, the negative impact of middle-aged abdominal obesity on cognitive function has been corroborated from multiple perspectives. Overweight and obesity in middle-aged adults are associated with an increased risk of incident cognitive decline, particularly in tasks related to executive function and memory (22). Abdominal obesity may also lead to brain structural abnormalities and metabolic dysfunction, both of which are significant risk factors for cognitive impairment and dementia (4). Obesity—particularly during middle age—not only adversely affects cognitive function but also elevates the risk of dementia in later life (23–25).

Collectively, population-based evidence consistently shows that midlife abdominal obesity is cross-sectionally associated with poorer global cognition, executive function, and memory across diverse populations. These associations are robust and reproducible (21, 22, 26). Longitudinal studies further confirm that higher baseline abdominal obesity predicts faster subsequent cognitive decline (8, 20, 21). The largest cohort studies report consistent effect sizes and directions, supporting the reliability of this association (4, 23–25). Although some inconsistent findings have been reported (27, 28), the overall convergence of cross-sectional and longitudinal evidence supports a genuine and robust link (21, 22, 26, 30–32). Population data from the CHARLS cohort indicate that 42% of middle-aged adults have elevated waist circumference and 25% show cognitive decline (29).

### 4.1 Evidence from cross-sectional studies

Cross-sectional studies consistently demonstrate an inverse association between abdominal obesity and cognitive performance in middle-aged populations. Table 1 summarizes key characteristics of representative cross-sectional studies included in this review. These findings collectively support a consistent negative association between central adiposity and cognitive function across diverse populations.

**Table 1:** Key characteristics of cross-sectional studies on abdominal obesity and cognitive function.

Study	Design	Population	Sample size (*n*)	Exposure	Key findings	TCM relevance
Chen et al. (33)	Cross-sectional	Middle-aged and older adults with type 2 diabetes	NR	WHRadjBMI	WHRadjBMI was associated with cognitive impairment	Not reported
Kesse-Guyot et al. (34)	Cross-sectional	French adults	NR	BMI, WC	Higher BMI and WC predicted lower executive function	Not reported
Ariyasinghe et al. (35)	Cross-sectional	Middle-aged Sri Lankans	NR	Obesity status	Obese adults scored lower on MoCA and MMSE (p <0.01)	Not reported
Bagya et al. (36)	Cross-sectional	Middle-aged women in South India	NR	Obesity	Gender-specific association between obesity and cognition	Not reported
Lentoor and Myburgh (37)	Cross-sectional	South African women (18–59 years)	175	BMI	BMI negatively correlated with MoCA scores	Not reported
Lu et al. (38)	Cross-sectional	Chinese adults ≥40 years (Beijing)	1,722	Obesity, glucose tolerance	Overweight/obese individuals had lower MMSE scores; central obesity associated with cognitive impairment	Not reported
Liu et al. (39)	Cross-sectional	Chinese middle-aged adults	NR	WC	Excessive WC was associated with poorer memory function	Not reported

BMI, body mass index; WC, waist circumference; WHRadjBMI, waist-to-hip ratio adjusted for body mass index; MoCA, Montreal Cognitive Assessment; MMSE, Mini-Mental State Examination; NR, not reported.

A cross-sectional study of middle-aged and older adults with type 2 diabetes found an association between adjusted waist-to-hip ratio (WHRadjBMI) and cognitive impairment (CI) (33), suggesting that abdominal obesity may be a risk factor for cognitive decline.

Cross-sectional studies conducted across diverse populations consistently indicate that abdominal obesity is associated with poorer executive function, memory, and global cognition. These associations are evident in Asian, European, and African cohorts, suggesting that the link between central adiposity and cognitive impairment may be generalizable across ethnic and geographic backgrounds (34). In Asian populations, middle-aged Sri Lankan adults with obesity exhibit significantly lower scores on MoCA and MMSE (*p* < 0.01) (35), while studies in South India highlight gender-specific patterns in the obesity–cognition relationship (36). A large-scale analysis of more than 5 million participants confirms that higher WC is associated with an increased risk of cognitive impairment (4). In African populations, a study among South African women demonstrates a significant negative correlation between BMI and MoCA scores (37).

Consistent findings from Chinese populations support the association between abdominal obesity and cognitive impairment. Population-based surveys show that overweight and obese middle-aged and older adults have lower cognitive test scores than normal-weight individuals, independent of impaired glucose metabolism and central obesity status (38). Excessive waist circumference is also correlated with poorer performance on memory function tests, further supporting the relationship between central adiposity and specific cognitive deficits (39).

Although cross-sectional studies cannot establish a definitive causal relationship between middle-aged abdominal obesity and cognitive function, they provide extensive epidemiological evidence supporting their association. Overall, cross-sectional evidence supports a robust association between central adiposity and cognitive impairment, although causal inference remains limited. Additionally, the use of different WC cut-offs across studies (e.g., Asian vs. Western criteria) and varying adjustment strategies complicates direct comparison of effect sizes across populations.

### 4.2 Evidence from longitudinal cohort studies

Longitudinal cohort studies provide stronger evidence supporting the temporal relationship between abdominal obesity and subsequent cognitive decline. Table 2 summarizes key characteristics of representative longitudinal cohort studies included in this review.

**Table 2:** Key characteristics of longitudinal cohort studies on abdominal obesity and cognitive function.

Study	Design	Population	Sample size (*n*)	Exposure	Duration	Key findings	TCM relevance
Wu et al. (21)	Cohort (CHARLS)	Chinese adults ≥45 years	10-year follow-up	Abdominal obesity	10 years	Abdominal obesity significantly associated with cognitive decline	Not reported
Rajan et al. (40)	Cohort (HRS)	Middle-aged adults	15,000	WC increase (10%)	10+ years	10% increase in WC → 20% faster cognitive decline; APOE ε4 carriers had higher risk	Not reported
Qureshi et al. (41)	Cohort	Middle-aged and older Europeans	>20,000	Metabolic abnormalities	Prospective	Greater metabolic abnormalities in midlife → higher dementia risk	Not reported
Hartanto et al. (42)	Cohort (MIDUS)	Middle-aged adults	2,652	WHR	9 years	Higher WHR → later declines in episodic memory; bidirectional relationship	Not reported
Zhang et al. (43)	Cohort (UK Biobank)	British adults	500,000	Obesity trajectories	Long-term	Stable or increasing obesity → impairments in brain morphology and cognition	Not reported

CHARLS, China Health and Retirement Longitudinal Study; HRS, Health and Retirement Study; MIDUS, Midlife in the United States; UK Biobank, United Kingdom Biobank; WC, waist circumference; WHR, waist-to-hip ratio; APOE ε4, apolipoprotein E ε4.

Data from the Health and Retirement Study (HRS) show that a 10% increase in waist circumference is associated with a 20% faster rate of cognitive decline, independent of age, education, and comorbidities (40). APOE ε4 carriers show greater vulnerability, indicating gene–environment interactions that may contribute to the underlying mechanism. A prospective European cohort of more than 20,000 participants demonstrates that midlife metabolic risk factors, including elevated waist circumference, are associated with higher dementia risk (41). The MIDUS study further supports a bidirectional relationship: higher baseline WHR predicts subsequent episodic memory decline, while improved baseline executive function is associated with lower later-life obesity risk (42).

A large-scale prospective cohort study using the UK Biobank (*n* = 500,000 British adults) analyzed genetic, lifestyle, health, and biological data (43). The analysis revealed that population trajectories characterized by increasing, moderately stable, and highly stable obesity were associated with progressively greater impairments in brain morphology, functional connectivity, and cognitive abilities (43). This further confirms the persistent negative impact of midlife obesity on cognitive function from a long-term developmental perspective. Collectively, longitudinal evidence strengthens the hypothesis that midlife abdominal obesity contributes to accelerated cognitive decline. Nevertheless, longitudinal studies cannot fully eliminate residual confounding, selective attrition, or the possibility that weight loss during prodromal neurodegeneration may precede the diagnosis of cognitive decline.

### 4.3 Stratified association analysis by abdominal obesity severity and cognitive outcome grade

#### 4.3.1 Stratification by abdominal obesity severity or exposure burden

Available study-level estimates generally support a graded relationship between greater adiposity burden and poorer cognitive outcomes. Each 0.27 kg increase in visceral fat corresponded to 0.7 years of cognitive aging (20); a 10% increase in WC was associated with a 20% faster rate of cognitive decline (40); and the UK Biobank trajectory analysis showed progressively greater brain and cognitive abnormalities across increasing or persistently high obesity trajectories (43). These contrasts support a dose- or duration-related interpretation, but they cannot be combined numerically because they represent different adiposity constructs, units, populations, and outcome models.

#### 4.3.2 Stratification by cognitive outcome grade

The association extends across a cognitive severity spectrum. At the earliest level, cross-sectional studies most often detected lower executive function, memory, processing speed, or global screening scores. Longitudinal studies then linked abdominal adiposity with accelerated decline or incident cognitive impairment, while large cohorts and contextual meta-analytic evidence extended the association to dementia and Alzheimer's disease. The reported 1-standard-deviation WC estimates were not strictly monotonic across grades: the relative estimate was higher for dementia (1.53) than for cognitive impairment (1.43), but lower for Alzheimer's disease (1.39) (4). This pattern does not refute an association across severity grades; rather, it shows that diagnostic specificity, competing risks, follow-up length, baseline age, and case ascertainment influence the measured effect. The available evidence therefore does not justify a simple proportional progression from subtle cognitive deficit to Alzheimer's disease.

#### 4.3.3 Joint interpretation and limitations of the stratified comparison

When adiposity severity and cognitive grade are considered jointly, the highest-risk pattern appears to involve persistent or increasing central adiposity combined with genetic susceptibility or metabolic comorbidity and followed longitudinally to domain-specific decline or clinical impairment. Nevertheless, most included studies did not simultaneously cross-classify participants by abdominal-obesity grade and cognitive-impairment grade, and participant-level data were unavailable. A formal interaction estimate, threshold analysis, or pooled comparison of correlation strength could therefore not be calculated. The findings below should be interpreted as a study-level dose-pattern synthesis rather than a subgroup meta-analysis, the quantitative contrasts discussed above are summarized in Table 3.

**Table 3:** Available quantitative contrasts informing the stratified association analysis.

Adiposity exposure contrast	Cognitive outcome grade	Reported association	Interpretation and comparability
Visceral fat increase of 0.27 kg	Continuous cognitive aging	Equivalent to 0.7 years of cognitive aging (20)	Supports a graded association with directly assessed visceral fat; not comparable with WC categories.
WC increase of 10%	Longitudinal cognitive decline	20% faster rate of cognitive decline (40)	Supports temporal and dose-related interpretation; a rate estimate cannot be pooled with risk ratios.
Increasing or persistently high obesity trajectory	Brain morphology, connectivity, and cognition	Progressively greater impairment across trajectories (43)	Supports cumulative exposure burden, but the trajectory reflects general rather than exclusively abdominal obesity.
Obesity vs. non-obesity	MoCA and MMSE performance	Lower scores in obese adults, *P* < 0.01 (35)	Supports a categorical difference in global cognition; cross-sectional design and broad obesity definition limit causal interpretation.
WC increase of 1 SD	Cognitive impairment	1.43-fold higher risk (4)	Contextual meta-analytic estimate; not treated as an original study-level effect.
WC increase of 1 SD	Dementia	1.53-fold higher risk (4)	Largest of the three contextual clinical estimates; latency and competing risks may influence magnitude.
WC increase of 1 SD	Alzheimer's disease	1.39-fold higher risk (4)	Supports disease-specific association but not a monotonic grade gradient; diagnostic specificity differs.

### 4.4 Evidence from intervention studies

Interventional studies offer preliminary evidence suggesting that reducing abdominal obesity may improve cognitive outcomes. Table 4 summarizes key characteristics of representative intervention trials.

**Table 4:** Key characteristics of intervention trials on abdominal obesity and cognitive function.

Study	Design	Population	Sample size (*n*)	Intervention	Control	Duration	Key findings	TCM relevance
Simpson et al. (44)	RCT	Adults with obesity (45–76 years)	5,145	Low-calorie diet + aerobic exercise	Standard care	6 months	Weight and WC reduced; cognitive scores improved by 10–15%	Not reported
Medawar and Witte (45)	Meta-analysis	Middle-aged adults with abdominal obesity	12 RCTs	Moderate-intensity weight loss interventions	Various	Variable	Pooled effect size (Cohen's d=0.4); intervention duration correlated with cognitive enhancement	Not reported

RCT, randomized controlled trial; WC, waist circumference.

The Look AHEAD (Action for Health in Diabetes) trial, a 6-month comprehensive weight loss intervention targeting middle-aged individuals with abdominal obesity and metabolic abnormalities, combined a low-calorie diet control with regular aerobic exercise. The intervention resulted in significant reductions in weight and waist circumference, and cognitive indicators, including executive function and information processing speed, improved by 10–15% relative to controls, demonstrating that reducing abdominal obesity may enhance cognitive function (43, 44).

A meta-analysis of 12 similar randomized controlled trials further supports these findings. With a pooled effect size (Cohen's d = 0.4), moderate-intensity weight management interventions significantly improve cognitive function in this population, with intervention duration positively associated with cognitive benefits (36, 45). These trials provide robust causal evidence that controlling abdominal obesity may delay cognitive decline, offering practical guidance for the development of clinical interventions.

In summary, cross-sectional, longitudinal, and interventional studies progressively build a comprehensive evidence chain linking middle-aged abdominal obesity to cognitive impairment, ranging from descriptive associations to causal inference and intervention validation. This foundation supports subsequent mechanistic research and clinical translation. However, current intervention evidence remains limited in scale and duration, highlighting the need for larger, long-term randomized trials. Interventional evidence therefore provides only secondary support for potential modifiability and should not be interpreted as definitive causal validation.

## 5 Potential biological mechanisms linking abdominal obesity to cognitive outcomes

**Figure 2:**
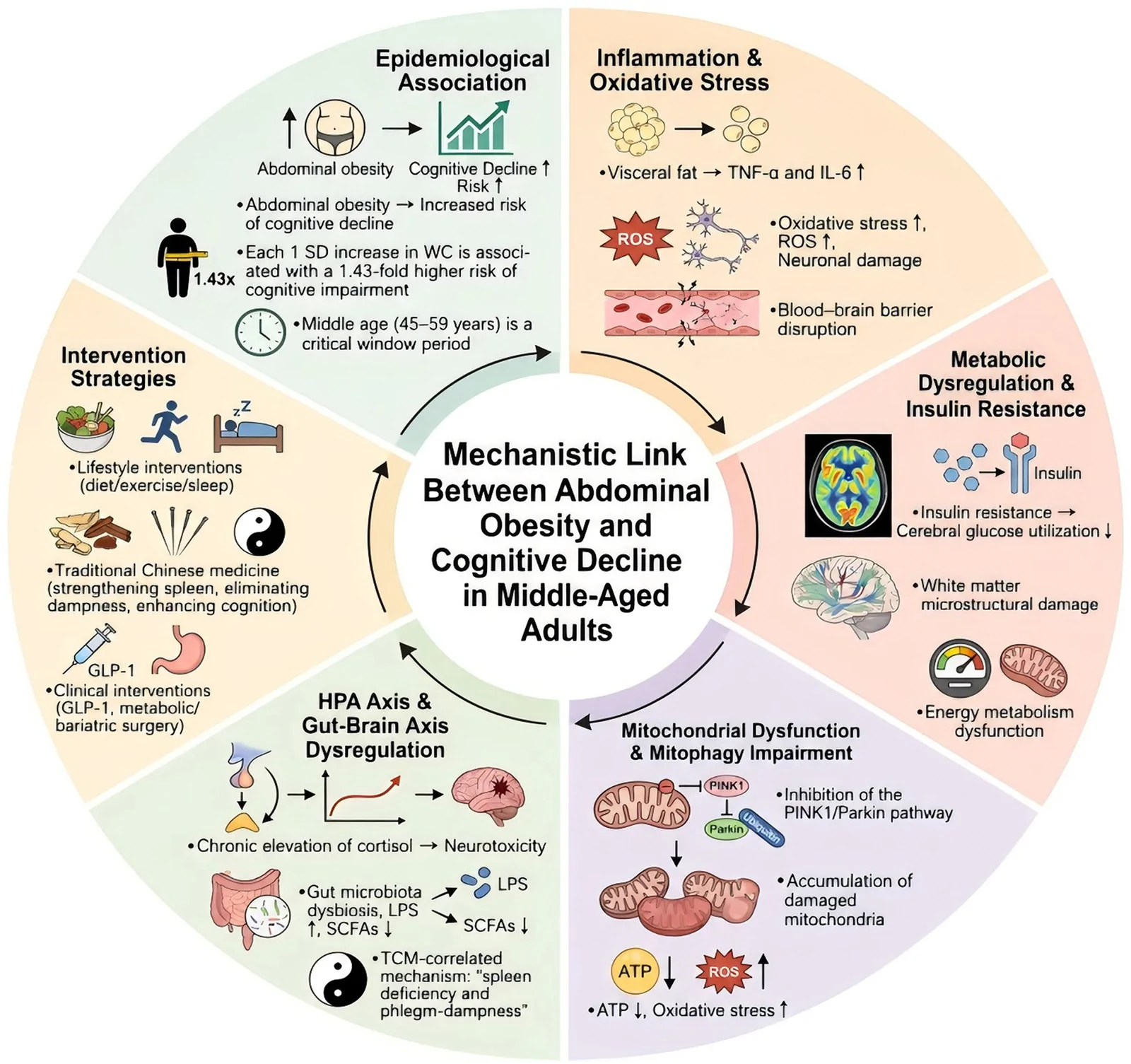
Mechanistic links between abdominal obesity and cognitive decline in middle-aged adults. Mechanistic links between abdominal obesity and cognitive decline in middle-aged adults. Abdominal obesity impairs cognitive function via interconnected, synergistic physiological pathways. Epidemiologically, visceral adiposity increases cognitive decline risk, with midlife representing a critical vulnerable window. Excess visceral fat drives chronic low-grade inflammation and oxidative stress, elevating pro-inflammatory cytokines (TNF-α, IL-6) and ROS to promote neuronal damage and blood-brain barrier disruption. Metabolic dysregulation, including insulin resistance, impairs cerebral glucose utilization and causes structural and functional brain injury. Mitochondrial dysfunction, marked by altered energy metabolism and impaired mitophagy, further exacerbates neuronal damage. HPA axis and gut-brain axis dysregulation involve chronic cortisol elevation, gut microbiota dysbiosis, and altered metabolites (reduced SCFAs, increased LPS), contributing to neurotoxicity and neuroinflammation. Lifestyle and clinical interventions target these pathways to mitigate abdominal obesity-related cognitive decline.

Midlife abdominal obesity may impair cognitive function through multiple interconnected physiological pathways (Figure 2). These pathways may not operate in isolation but may interact synergistically to accelerate cognitive decline. The specific mechanisms are as follows:

Abdominal obesity is associated with an increased risk of cognitive decline in middle-aged adults. Multiple interconnected pathways are involved, including chronic inflammation and oxidative stress, insulin resistance and metabolic dysregulation, mitochondrial dysfunction and impaired mitophagy, as well as hypothalamic-pituitary-adrenal (HPA) axis and gut-brain axis dysregulation. These pathways collectively mediate the adverse impacts of abdominal obesity on cognitive function.

### 5.1 Inflammation and oxidative stress pathways

In middle-aged individuals with abdominal obesity, adipose tissue—particularly visceral fat—releases pro-inflammatory factors such as tumor necrosis factor-α (TNF-α) and interleukin-6 (IL-6), which may contribute to systemic inflammation (6). These factors may enter the central nervous system through the bloodstream, potentially inducing neuroinflammation and compromising blood-brain barrier integrity, leading to neuronal damage. These cytokines may inhibit hippocampal neurogenesis, contributing to memory-related cognitive decline (46). Concurrently, inflammation is accompanied by heightened oxidative stress, where excessive reactive oxygen species (ROS) may further exacerbate neuronal oxidative damage, potentially forming a vicious cycle that persistently impairs cognitive function.

### 5.2 Insulin resistance and metabolic dysregulation pathways

Abdominal obesity in middle age is often accompanied by insulin resistance and hyperinsulinemia, potentially causing systemic metabolic disorders (47). Within the central nervous system, insulin resistance may reduce cerebral glucose uptake and utilization, impairing synaptic plasticity and signal transmission, potentially contributing to cognitive decline (48). Significant correlations between insulin resistance and white matter microstructural lesions have been observed, suggesting that metabolic dysfunction may impair cognition by altering white matter integrity (49).

### 5.3 Neuroendocrine axis dysregulation pathways

Middle-aged abdominal obesity may activate the hypothalamic-pituitary-adrenal (HPA) axis, potentially leading to chronic cortisol elevation (50). Prolonged hypercortisolemia may exert neurotoxic effects, particularly on the prefrontal cortex—a brain region responsible for executive function—potentially disrupting neuronal signaling. Significant positive correlations between elevated cortisol levels and executive function decline have been confirmed in human studies, suggesting that neuroendocrine dysregulation may mediate the relationship between middle-aged abdominal obesity and cognitive impairment (51).

### 5.4 Mitochondrial dysfunction and impaired mitophagy pathways

Metabolic disorders induced by abdominal obesity may inhibit mitophagy in neuronal mitochondria (52). Mitochondria are core organelles for cellular energy metabolism; impaired function, such as excessive ROS production and reduced adenosine triphosphate (ATP) synthesis, may directly lead to neuronal dysfunction (53). Free fatty acids from visceral fat may disrupt the mTOR-PINK1/Parkin signaling pathway, thereby impairing the clearance of damaged mitochondria (54, 55). Reduced mitochondrial clearance efficiency in the hippocampus may exacerbate oxidative stress and energy defects, ultimately potentially leading to cognitive impairment (56, 57).

### 5.5 Gut-brain axis disturbance pathways

Emerging evidence highlights the role of the gut-brain axis in linking abdominal obesity to cognitive impairment (58). The gut microbiota of individuals with abdominal obesity may exhibit reduced diversity and dysbiosis, characterized by an increased Firmicutes/Bacteroidetes ratio. This dysbiosis may increase intestinal permeability (“leaky gut”), allowing lipopolysaccharides (LPS) to enter the circulation and trigger systemic inflammation and neuroinflammation (59, 60).

Concurrently, gut microbiota regulate short-chain fatty acids (SCFAs), such as butyrate, which have neuroprotective effects. Obesity-related dysbiosis may reduce SCFA production, potentially impairing blood-brain barrier integrity and promoting neuroinflammation (61, 62). Furthermore, the gut-brain axis may interact closely with the HPA axis: dysbiosis may stimulate HPA axis activity, potentially increasing cortisol levels, which in turn may exacerbate both abdominal obesity and cognitive decline (63).

### 5.6 Cross-pathway amplification and feed-forward interactions

The pathways described above do not operate independently but form an interconnected, self-amplifying network. Visceral adipose tissue-derived free fatty acids and inflammatory cytokines promote insulin resistance, which in turn impairs cerebral glucose metabolism and compromises mitophagy, leading to ROS accumulation and neuronal damage. Concurrently, obesity-induced gut dysbiosis increases intestinal permeability, enabling LPS translocation that activates TLR4 signaling and amplifies neuroinflammation while overstimulating the HPA axis. The resulting cortisol elevation promotes further visceral fat deposition and prefrontal neurotoxicity. This feed-forward loop explains why moderate abnormalities across multiple pathways may produce greater cognitive effects than a single severe defect.

## 6 Modifying factors

**Figure 3:**
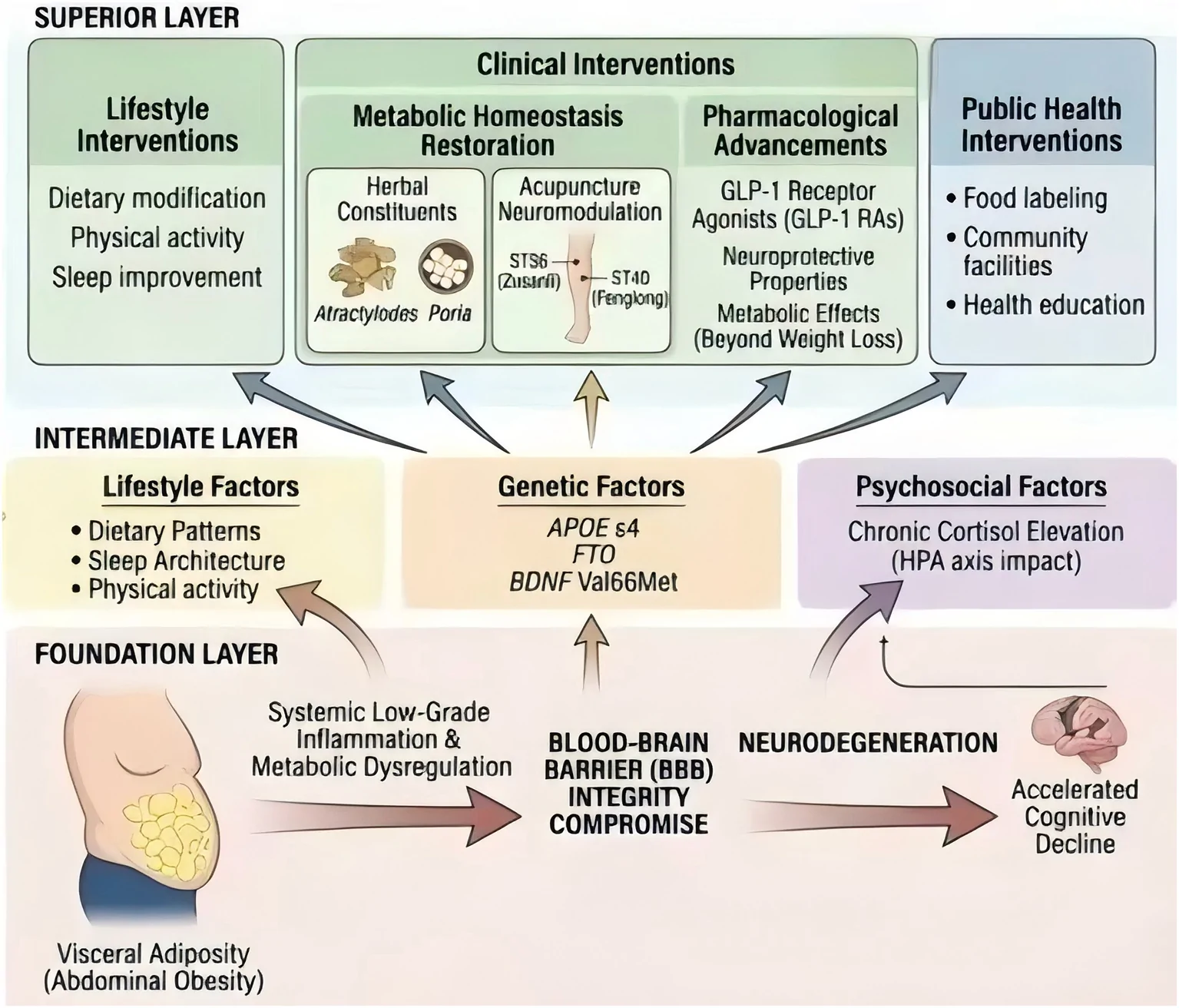
Integrated framework of influencing factors and intervention strategies for abdominal obesity-related cognitive decline. This figure illustrates the multi-layered framework of factors influencing the association between midlife abdominal obesity and cognitive decline, as well as corresponding intervention strategies. At the foundation layer, visceral adiposity drives systemic low-grade inflammation, metabolic dysregulation, blood-brain barrier disruption, and neurodegeneration, leading to accelerated cognitive decline. The intermediate layer includes modifiable lifestyle factors, genetic predispositions, and psychosocial stressors that modulate this relationship. The superior layer presents multi-modal interventions targeting these pathways, including lifestyle modifications, metabolic restoration, pharmacological advancements, and public health strategies, forming a comprehensive translational framework.

The association between middle-aged abdominal obesity and cognitive function may be complexly modulated by multidimensional factors—including lifestyle, genetic background, and psychosocial status—which may interact to amplify or attenuate its impact (Figure 3). Their core mechanisms may be closely linked to metabolic disorders, inflammatory responses, and neurofunctional impairment, as detailed below.

### 6.1 Lifestyle factors

Lifestyle factors play a critical role in modulating the relationship between abdominal obesity and cognitive function. Dietary patterns, physical activity, and sleep quality interact to influence both metabolic health and cognitive outcomes, offering modifiable targets for intervention.

#### 6.1.1 Dietary patterns

The impact of dietary patterns on brain health has emerged as a significant research focus, particularly among middle-aged and elderly populations who face heightened risks of cognitive decline and neurodegenerative diseases (64). Dietary patterns may directly influence abdominal obesity and cognitive function by regulating energy metabolism, inflammatory responses, and gut-brain axis function.

Westernized diets rich in sugar, fat, and highly processed foods may exacerbate metabolic burdens and visceral fat deposition, and may further impair cognitive function by intensifying systemic inflammation and disrupting gut microbiota balance. High-sugar diets may induce severe blood glucose fluctuations, potentially inhibiting hippocampal synaptic plasticity (65). High-fat diets may trigger insulin resistance, potentially damaging neuronal survival and insulin-sensitivity pathways, thereby affecting synaptic plasticity (66). Human studies have confirmed a significant positive correlation between excessive intake of high-fat, high-sugar diets and reduced hippocampal-related memory (66).

Conversely, diets rich in omega-3 fatty acids, fiber, and antioxidants—exemplified by the Mediterranean pattern—may mitigate cognitive decline and reduce abdominal obesity through anti-inflammatory and metabolic-improving mechanisms (67–70). Better dietary quality in midlife is associated with enhanced functional connectivity between the hippocampus and occipital lobe/cerebellum in later life, as well as preserved white matter structural integrity: higher fractional anisotropy (FA) and lower mean/axial diffusivity (MD/AD) in regions including the corpus callosum and thalamic radiations (71). Consistent protective associations between dietary quality and cognitive function have also been observed in Chinese adults (72).

Collectively, these findings identify midlife as a critical window for dietary intervention: improving dietary quality may simultaneously alleviate metabolic dysfunction, visceral fat deposition, and subsequent cognitive decline.

#### 6.1.2 Physical activity

Physical activity may act as a crucial intervention to regulate the association between abdominal obesity and cognitive function by modulating energy expenditure, improving insulin sensitivity, and promoting neurotrophic factor secretion. Systematic reviews and meta-analyses confirm that sedentary behavior and insufficient physical activity (less than 150 min of exercise per week) both increase the risk of obesity (73) and may significantly amplify the association between midlife abdominal obesity and cognitive impairment (74, 75).

Sedentary behavior may reduce energy expenditure, promote visceral fat accumulation, and potentially decrease cerebral blood flow, thereby lowering oxygenation in cognition-related regions such as the prefrontal cortex and hippocampus (47). Prolonged sitting may reduce cerebral perfusion and impair executive function, while visceral fat accumulation may further exacerbate cerebral blood flow deprivation in these regions (76, 77). The prefrontal cortex and hippocampus govern core cognitive functions, including planning, decision-making, working memory, and memory formation, so impairment in either region may directly contribute to cognitive decline (78–81).

Regular moderate-to-high-intensity exercise (e.g., brisk walking or swimming for 45 min three times weekly) may mitigate the negative cognitive effects of abdominal obesity by promoting lipolysis and improving insulin sensitivity (82). Concurrently, exercise may increase secretion of brain-derived neurotrophic factor (BDNF), a key mediator of neuroprotection and synaptic plasticity that supports neuronal repair and enhances executive function and memory (83, 84). Higher cardiorespiratory fitness, muscle strength, and healthy weight are consistently associated with better cognitive function in middle-aged individuals, further supporting exercise's protective role against obesity-related cognitive impairment (85).

#### 6.1.3 Sleep quality

Sleep quality may influence the association between abdominal obesity and cognitive function by regulating hormonal balance, metabolic waste clearance, and neuroinflammatory responses. Chronic sleep deprivation (less than 6 h per night) or obstructive sleep apnea (OSA) may disrupt hormonal equilibrium, potentially increasing abdominal obesity risk and accelerating neurodegenerative changes (86). Pathologically, sleep deprivation may disrupt the balance of appetite-regulating hormones leptin and ghrelin, reducing leptin levels while increasing ghrelin levels (87, 88). This hormonal imbalance may increase appetite, particularly cravings for high-carbohydrate and high-fat foods, leading to excessive calorie intake, reduced energy expenditure, and subsequent weight gain and abdominal obesity (89). Consistent with this, leptin dysregulation has been observed in both sleep-deprived adults and patients with obstructive sleep apnea (88, 89). Concurrently, obesity—a primary risk factor for OSA—may exacerbate metabolic disorders through intermittent hypoxia, sympathetic nervous system activation, and systemic inflammation, potentially forming a vicious cycle of “obesity-OSA” (90).

Additionally, sleep deprivation may reduce slow-wave sleep duration—a critical phase for the brain's clearance of metabolic waste products such as β-amyloid. The synergistic effect of these factors may accelerate neurodegenerative changes, potentially increasing the risk of cognitive impairment in individuals with abdominal obesity (91).

### 6.2 Genetic factors

Genetic factors may influence the strength of the association between midlife abdominal obesity and cognitive function by regulating fat distribution, metabolic efficiency, and neuroprotective capacity. While relevant genes may not directly determine cognitive outcomes, they may amplify or mitigate the cognitive impact of abdominal obesity through interactions with environmental factors (e.g., diet, exercise).

#### 6.2.1 Apolipoprotein E ε4 (APOE ε4)

The apolipoprotein E ε4 (APOE ε4) allele may exhibit complex interactions with obesity and cognitive function (92). Its presence may exacerbate the negative impact of high saturated fat intake on cognitive function, suggesting that gene-diet interactions may accelerate cognitive decline (93).

#### 6.2.2 Fat mass and obesity-associated gene (FTO)

The FTO gene is a key gene regulator of fat metabolism. Middle-aged individuals carrying the FTO risk allele may accumulate visceral fat at a rate 1.8 times faster than non-carriers (94) and may exhibit reduced sensitivity to brain insulin signaling pathways (95). This mechanism is consistent with the insulin resistance observed in abdominal obesity, thus increasing vulnerability to obesity-related cognitive decline.

#### 6.2.3 Brain-derived neurotrophic factor (BDNF) Val66Met polymorphism

Individuals with abdominal obesity carrying the BDNF Met allele may exhibit reduced BDNF secretion, diminished neuronal repair capacity, and a significantly increased risk of cognitive impairment (96). Reduced hippocampal BDNF expression, as observed in high-fat diet-induced obesity models in rats, further supports the association between obesity and cognitive dysfunction (97).

### 6.3 Psychosocial factors

Psychosocial factors may indirectly influence the relationship between midlife abdominal obesity and cognitive function by regulating stress responses and behavioral choices. Core factors include chronic stress and social support.

#### 6.3.1 Chronic stress

Prolonged chronic stress (e.g., excessive workloads, financial pressures, negative family events) may activate the HPA axis, potentially leading to sustained cortisol elevation. This may simultaneously promote visceral fat synthesis and exacerbate abdominal obesity, while potentially directly damaging prefrontal cortex neurons and diminishing executive function (decision-making, attention regulation) (98).

Depression, a significant manifestation of chronic stress, may exhibit synergistic effects with abdominal obesity on cognitive function in middle-aged and elderly populations. Individuals with both depressive stress and abdominal obesity face a significantly higher risk of cognitive impairment than those with only one risk factor (99). Multiple psychosocial stressors are associated with lower baseline cognitive function and accelerated cognitive decline in middle-aged and older adults (100), and higher cognitive impairment prevalence is observed among individuals with abdominal obesity in high-stress compared to low-stress groups (101). Collectively, these findings suggest that chronic stress may impair cognitive function synergistically with abdominal obesity.

#### 6.3.2 Social support

Social support (including family care, peer assistance, and community involvement) may play a protective role in the association between abdominal obesity and cognitive function by buffering stress and promoting healthy behaviors. From a behavioral regulation perspective, middle-aged individuals with adequate social support are more likely to adhere to healthy lifestyle habits such as regular diet and exercise, thereby achieving better control of abdominal obesity (81). Higher social support scores correlate with lower BMI and waist circumference (WC) (102), and even among socially isolated obese and overweight adults, social support remains a key factor in promoting healthy behavior change via social media and social cognitive theory (103).

From a neuroprotective perspective, active social interaction may directly stimulate cognitive activity and reduce neuroinflammation caused by loneliness (81). Longitudinal data from Chinese older adults indicate that changes in social engagement may influence cognitive function (104), with positive neighborhood interactions exerting beneficial cognitive effects (105). Social support may also indirectly protect cognitive function by modulating the impact of AD-related neurodegenerative changes on cognitive decline (106) and by positively affecting psychological wellbeing, mediated through cognitive flexibility (107).

Lifestyle, genetic, and psychosocial factors interact synergistically to modulate the association between abdominal obesity and cognitive decline in middle-aged adults. Dietary patterns, physical activity, and sleep independently and jointly influence metabolic health, inflammation, and neuroplasticity. Genetic polymorphisms, including APOE ε4, FTO, and BDNF, further shape individual vulnerability, while chronic stress amplifies risk through neuroendocrine and metabolic disturbances. These factors do not act in isolation but act in concert to significantly increase the risk of cognitive impairment.

Therefore, interventions for middle-aged populations should focus on lifestyle and psychosocial characteristics, targeting key pathways such as inflammation, insulin resistance, and mitochondrial function. Strengthening the coordinated regulation of modifiable factors may achieve dual objectives: obesity management and cognitive protection.

## 7 Intervention evidence and implications

Because the primary purpose of this review is to synthesize the association between abdominal obesity and cognitive outcomes and their underlying mechanisms, intervention evidence is summarized only to define the limits of clinical translation rather than to screen or rank treatments.

### 7.1 Lifestyle interventions

Lifestyle modification may require synergistic optimization of diet, exercise, and sleep. Dietary approaches described in the included studies include a “low glycemic index + high nutrient density” pattern limiting saturated and trans fats while increasing intake of omega-3 fatty acids, dietary fiber, and antioxidants, thereby potentially regulating gut microbiota composition and reducing neuroinflammation (69–72).

Exercise protocols described in the included studies involve 150 min of moderate-intensity aerobic exercise weekly combined with 2 sessions of resistance training. For individuals with MCI, cognitive-motor integration training such as Tai Chi or square dancing has been suggested, as it may enhance neuronal plasticity by stimulating BDNF secretion (83, 84).

Sleep interventions examined in the included studies focus on structural optimization, including regular sleep routines and regulating sleep environment temperature. Patients with moderate to severe OSA may require continuous positive airway pressure (CPAP) therapy to potentially reduce the accumulation of metabolic waste in the brain (86).

### 7.2 Pharmacological interventions

Clinical interventions have been studied in middle-aged individuals with moderate to severe abdominal obesity (BMI ≥28 kg/m^2^) or those with evidence of cognitive decline who do not respond adequately to lifestyle modifications. Such interventions are generally implemented under medical supervision.

Western medicine currently lacks specific drugs targeting “abdominal obesity-related cognitive decline.” Glucagon-like peptide-1 (GLP-1) receptor agonists may indirectly affect cognitive pathways by reducing visceral fat, but they are associated with gastrointestinal side effects, and evidence for direct cognitive benefits remains limited (108, 109). Cholinesterase inhibitors may only alleviate symptoms and may not reverse obesity-induced neural damage; long-term use may increase hepatic and renal burdens (110).

### 7.3 Metabolic and bariatric surgery

Metabolic surgery (e.g., laparoscopic sleeve gastrectomy) may be considered in selected cases of severe abdominal obesity (typically BMI ≥32.5 kg/m^2^). This approach may rapidly reduce body weight and visceral fat, potentially improving cerebral blood flow and cognitive function. However, risks include infection and malnutrition, necessitating strict indication control (111).

### 7.4 Traditional Chinese medicine-related approaches

TCM interventions, including herbal formulations, acupuncture, and acupoint catgut embedding, have been explored as complementary strategies based on “invigorating the spleen and resolving dampness” frameworks.

Preliminary evidence suggests potential effects on inflammation, gut microbiota, and the HPA axis. However, current findings are mainly from preclinical or small-scale studies, with unclear mechanisms and insufficient high-quality randomized controlled trials.

Thus, TCM-related interventions remain exploratory complementary strategies. Further rigorous research is needed to confirm clinical benefits and underlying mechanisms.

## 8 Discussion

### 8.1 Summary of main findings

This systematic review synthesized evidence from 96 studies examining the association between abdominal obesity and cognitive outcomes in middle-aged adults. Overall, the available evidence consistently indicates that central adiposity, commonly assessed by waist circumference, waist-to-hip ratio, or related measures, is associated with poorer cognitive performance and an increased risk of cognitive decline in midlife populations (4, 8, 112).

#### 8.1.1 Association between abdominal obesity and cognitive outcomes

The included studies strongly support midlife abdominal obesity as an independent risk factor for cognitive impairment and decline. This association is evident for both global cognition and specific domains such as memory and executive function. However, effect magnitudes vary considerably across study designs: the meta-analysis of >5 million participants reported an OR of 1.43 per 1-SD WC increase for cognitive impairment, whereas individual cohort studies show a wider range (OR: 1.2–2.5). Longitudinal studies generally yield larger estimates than cross-sectional designs, and studies using imaging-based visceral fat measures report stronger associations than those using simple anthropometry, suggesting that measurement precision meaningfully influences observed effect sizes (10, 21, 113, 114).

#### 8.1.2 Interaction of multiple biological pathways

The biological basis is multifactorial and interconnected, but critically, these pathways form a hierarchically organized, self-amplifying network rather than acting independently. Visceral adiposity-derived FFAs and pro-inflammatory cytokines (TNF-α, IL-6) directly impair peripheral and central insulin signaling, reducing cerebral glucose uptake and compromising mitophagy (e.g., via the PINK1/Parkin pathway). This leads to ROS accumulation and hippocampal synaptic damage. Simultaneously, gut dysbiosis increases intestinal permeability, enabling LPS translocation that activates microglial TLR4 signaling, amplifying neuroinflammation while overstimulating the HPA axis. The resulting cortisol elevation promotes further visceral fat deposition and prefrontal neurotoxicity. Thus, dysfunction in any single node—metabolic, inflammatory, mitochondrial, or neuroendocrine—reinforces the others through feed-forward loops and moderate abnormalities across multiple pathways may be more harmful than a severe defect in a single pathway, driving cognitive decline synergistically (6, 48, 52, 58).

#### 8.1.3 Modifiers, mediators, confounders, and bidirectionality

Diet, physical activity, sleep, genetic susceptibility, and psychosocial stress do not all play the same analytical role. Lifestyle factors may precede central adiposity, mediate part of its effect, or influence cognition independently; genetic variants such as APOE ε4 are more plausibly effect modifiers; and depression, stress, and social isolation may be both causes and consequences of adverse metabolic behavior. Adjustment for these variables can therefore either reduce confounding or remove part of the pathway of interest. This helps explain why fully adjusted models may report smaller or inconsistent associations even when the underlying biological relationship is similar (74, 93, 95, 97).

#### 8.1.4 Intervention evidence as supplementary causal support

Intervention studies suggest lifestyle-based strategies may confer dual benefits. However, current evidence is largely derived from short-term trials ( ≤ 12 months). Preliminary results are encouraging—dietary modification, physical activity, and sleep optimization reduce abdominal obesity measures and show concurrent cognitive improvements in some studies—but long-term sustainability remains unconfirmed. These findings suggest promising directions for future research and clinical consideration, though their long-term efficacy and generalizability remain to be established (44, 45).

Taken together, the current evidence supports a biologically plausible association between abdominal obesity and adverse cognitive outcomes in midlife. However, substantial heterogeneity in study design, exposure assessment, and cognitive evaluation frameworks limits direct comparability across studies and precludes definitive causal conclusions. Importantly, the consistency of findings across epidemiological, mechanistic, and preliminary interventional evidence strengthens the hypothesis that midlife central adiposity may represent a modifiable target for early prevention of cognitive decline.

### 8.2 Clinical and public health implications

A key implication of this review is the identification of midlife as a critical window for intervention. Unlike older adults, in whom cognitive decline may already reflect established neurodegenerative pathology, middle-aged individuals with abdominal obesity may still be within a potentially reversible stage of metabolic and neurobiological dysfunction.

#### 8.2.1 The importance of midlife as a critical window for intervention

Middle age is a pivotal stage for preventing cognitive decline, as metabolic disturbances may still be corrected before irreversible neuropathological changes occur. Thus, early interventions targeting midlife abdominal obesity hold great public health potential to delay or prevent late-life neurodegenerative diseases (4, 115).

#### 8.2.2 The potential of abdominal obesity assessment as an early indicator of cognitive vulnerability

From a public health perspective, routine assessment of abdominal obesity should not be limited to cardiometabolic risk evaluation but may also serve as an early indicator of cognitive vulnerability. Integrating simple anthropometric measures such as waist circumference with cognitive screening tools could facilitate earlier identification of at-risk individuals (21, 29).

#### 8.2.3 Population-level strategies targeting modifiable lifestyle factors

From a public health standpoint, population-level strategies targeting modifiable lifestyle factors—including dietary quality, physical activity, and sleep patterns—may offer a cost-effective approach to concurrently alleviate the burden of central obesity and mitigate long-term cognitive decline. Importantly, intervening on abdominal obesity during midlife may represent a strategic opportunity to delay or prevent later-life neurodegenerative diseases (70, 74, 87).

Notably, such public health strategies may be particularly impactful in midlife, as metabolic disturbances remain responsive to modification before irreversible brain damage occurs. Targeting abdominal obesity in midlife may provide a valuable opportunity to prevent or delay cognitive decline later in life.

However, rolling out such integrated strategies requires further evidence regarding long-term effectiveness and cost-efficiency, particularly across diverse populations and healthcare settings.

### 8.3 Heterogeneity and inconsistent evidence

#### 8.3.1 Exposure definition and adiposity-severity heterogeneity

WC, WHR, WHtR, visceral adiposity index, and imaging-based visceral fat are related but not equivalent. WC reflects both subcutaneous and visceral abdominal tissue and varies with stature and sex; WHR is influenced by both waist and hip circumference; and imaging measures more directly quantify visceral fat but are used in smaller and more selected samples. Ethnic- and sex-specific cut-offs, continuous vs. categorical modeling, and adjustment for BMI can therefore produce different effect sizes even when the underlying population relationship is similar.

#### 8.3.2 Cognitive measurement and impairment-grade heterogeneity

Cognitive outcomes ranged from brief global screens to domain-specific tests, change scores, MCI, dementia, and Alzheimer's disease. MMSE and MoCA may show ceiling effects in midlife and are sensitive to education, language, and cultural context; domain-specific tests can detect subtler deficits but yield less directly comparable scales. Clinical diagnoses require longer follow-up and may use different criteria. Consequently, inconsistent magnitude across cognitive grades may reflect outcome sensitivity and ascertainment rather than a true biological contradiction.

#### 8.3.3 Population, confounding, and analytical heterogeneity

Study populations differed in age span, sex distribution, menopausal status, ethnicity, diabetes and vascular disease burden, depression, sleep apnea, education, and socioeconomic status. These variables may confound or modify the association. Analytical choices also matter: minimally adjusted models may overestimate the central-adiposity effect through residual confounding, whereas models adjusting for diabetes, hypertension, inflammation, physical activity, or depression may underestimate a total effect by controlling for mediators. APOE-related vulnerability and sex-specific findings further argue against assuming a single common effect.

#### 8.3.4 Design-related inconsistency and observational-interventional discordance

Cross-sectional studies are vulnerable to reverse causality, longitudinal studies to attrition, exposure misclassification, and prodromal weight change, and Mendelian randomization studies to instrument validity and pleiotropy. Intervention studies may show smaller or inconsistent cognitive effects despite strong observational associations because exposure duration before treatment is long, some neural injury may be only partly reversible, follow-up is short, and multicomponent interventions do not specifically target visceral fat. Practice effects and secondary cognitive endpoints can further obscure change. Thus, observational and interventional evidence answer different questions and should not be expected to yield identical effect sizes.

Taken together, current evidence robustly supports an epidemiological association between abdominal obesity and adverse cognitive outcomes in midlife, whereas mechanistic and interventional conclusions remain comparatively less definitive. Therefore, although the overall relationship appears biologically plausible and clinically relevant, further high-quality prospective studies and rigorously designed randomized controlled trials are required to strengthen causal inference and translational applicability (Table 5).

**Table 5:** Evidence hierarchy, consistency, and interpretive limitations regarding abdominal obesity and cognitive outcomes.

Evidence category	Relative evidentiary value	Association-focused interpretation	Principal limitations	Priority
Longitudinal cohort studies	Moderately strong	Support temporal association, cumulative exposure, and cognitive decline	Residual confounding, attrition, prodromal weight change	Repeated exposure and cognitive measures
Cross-sectional studies	Moderate for coexistence	Consistent inverse association, especially for executive function and memory	No temporal direction; reverse causality	Domain-sensitive tests and causal models
Mendelian randomization studies	Potentially informative but limited	May test causal liability to adiposity	Instrument validity, pleiotropy, phenotype mismatch	Central-adiposity-specific instruments
Mechanistic studies	Biologically coherent	Support interacting inflammatory, metabolic, neurovascular, mitochondrial, HPA, and gut-brain pathways	Limited simultaneous human validation	Longitudinal mediation studies
Lifestyle interventions	Supplementary	Suggest potential modifiability but do not isolate WC reduction	Multicomponent exposure, short duration, secondary cognitive endpoints	Prespecified mediation and cognitive outcomes
Pharmacological and surgical evidence	Limited and indirect	Contextual metabolic evidence only	Selected populations and small cognitive evidence base	Mechanistic cognitive endpoints
TCM-related evidence	Exploratory	Hypothesis-generating only	Predominantly preclinical or small studies	Rigorous trials only if pursued

### 8.4 Sources of heterogeneity and inconsistent evidence

Despite overall consistency, heterogeneity across studies warrants examination. Three primary sources were identified. First, exposure metrics: WC thresholds differ between Asian (≥90 cm for men) and Western (≥102 cm) criteria, directly affecting comparability of risk estimates. Second, cognitive assessments: MoCA-based studies reported stronger associations than MMSE-based studies, attributable to MoCA's higher sensitivity for mild impairment. Third, covariate adjustment: studies adjusting for education, physical activity, and baseline cognition showed attenuated effect sizes, indicating these variables act as both confounders and effect modifiers. The few null findings [e.g., Chen et al. (27); Dye et al. (28)] involved non-standard exposures (VAI) or substantial between-study heterogeneity, and do not undermine the overall evidence. These observations underscore the need for methodological standardization in future research.

### 8.5 Evidence hierarchy, strengths, and limitations of the current review

This review has several strengths: it focuses specifically on abdominal rather than general obesity, integrates cross-sectional, longitudinal, mechanistic, Mendelian randomization, and intervention evidence, and explicitly compares available study-level estimates across exposure severity and cognitive outcome grades. Nevertheless, several limitations should be acknowledged.

#### 8.5.1 Limitations of causal inference and reverse causality

The majority of included studies are observational in design, which limits causal inference. Although longitudinal studies provide temporal evidence, reverse causality cannot be excluded, as early cognitive decline may influence lifestyle behaviors and contribute to the development of obesity (21, 42, 116, 117). Mendelian randomization studies are needed to better disentangle the causal relationship between abdominal obesity and cognitive decline (118).

#### 8.5.2 Influence of residual confounding factors

Residual confounding remains a concern. Factors such as socioeconomic status, educational attainment, comorbidities, and lifestyle behaviors may not have been fully controlled across studies, potentially influencing observed associations (119).

#### 8.5.3 High heterogeneity in exposure assessment and outcome measurement

Substantial heterogeneity exists in exposure assessment and outcome measurement. Different studies employed varying definitions of abdominal obesity (e.g., WC, WHR, WHtR, imaging-based measures) and cognitive outcomes (e.g., MMSE, MoCA, domain-specific tests), which may limit comparability and preclude quantitative synthesis (4). Finally, while emerging evidence suggests potential roles for complementary approaches such as traditional Chinese medicine (TCM), these findings should be interpreted cautiously. TCM-related interpretations are considered exploratory and hypothesis-generating rather than evidence-confirmed, and further high-quality randomized controlled trials are warranted to validate their efficacy.

### 8.6 Future research directions

Future research should prioritize precise characterization of the association, its dose-response form, temporal direction, and interacting mechanisms. Intervention trials are useful mainly as secondary tests of causal reversibility rather than as the principal research line.

#### 8.6.1 Use repeated adiposity measures with domain-specific cognition, neuroimaging, and biomarkers

Prospective studies should repeatedly measure WC, WHtR, and, in subsamples, imaging-derived visceral fat, alongside executive function, memory, processing speed, and global cognition (120–122). Structural and functional MRI, DTI, PET, and inflammatory, metabolic, vascular, and neurodegenerative biomarkers can determine whether changes in central adiposity precede changes in brain structure and whether proposed mediators track the same temporal sequence (123, 124).

#### 8.6.2 Distinguish effect modification from mediation using genetic, metabolic, and lifestyle data

Future analyses should prespecify causal models that distinguish confounders, mediators, and effect modifiers. APOE ε4, FTO, and BDNF variants, sex, menopausal status, diabetes, sleep apnea, depression, and lifestyle patterns should be evaluated through interaction and mediation analyses rather than being entered indiscriminately into fully adjusted models (125–127).

#### 8.6.3 Standardize abdominal-obesity severity and cognitive-impairment grades

Studies should report harmonized WC and visceral-fat categories, continuous dose-response estimates, and clinically interpretable increments. Cognitive outcomes should be separated into domain-specific performance, global impairment or MCI, and dementia or Alzheimer's disease. Cross-classified reporting of adiposity grade by cognitive grade would permit formal interaction, threshold, and trajectory analyses that were not possible in the present review. Furthermore, comprehensive intervention strategies targeting multiple pathways—combining dietary modification, physical activity, and cognitive training—should be evaluated in parallel to achieve more significant and long-lasting cognitive benefits (45, 128, 129).

#### 8.6.4 Validate association patterns across populations and use interventions sparingly for causal testing

Multi-center studies should examine whether severity gradients and affected cognitive domains differ by sex, ethnicity, region, socioeconomic context, and cardiometabolic background (130). Carefully designed interventions may then test whether reducing visceral adiposity changes prespecified cognitive and mechanistic endpoints, but such trials should be framed as causal validation of the association rather than as broad treatment screening.

## 9 Conclusion

Abdominal obesity is consistently associated with poorer cognitive outcomes in middle-aged adults, with evidence spanning domain-specific deficits, accelerated decline, cognitive impairment, dementia, and Alzheimer's disease. Available continuous and trajectory-based estimates are compatible with greater risk at higher or more persistent adiposity exposure, but heterogeneous exposure measures and non-uniform cognitive grades prevent a definitive pooled dose-response conclusion. The biological relationship is best explained by interacting inflammatory, insulin-signaling, neurovascular, neuroendocrine, mitochondrial, and gut-brain pathways that can amplify one another over time. Lifestyle, genetic, metabolic, sleep, and psychosocial factors may act as confounders, mediators, or effect modifiers and partly account for inconsistent findings. Lifestyle interventions show promise for improving both metabolic and cognitive outcomes, whereas pharmacological, surgical, and complementary approaches require further validation in large, well-designed RCTs. Future prospective studies should standardize severity categories, use repeated and domain-sensitive cognitive assessments, and integrate imaging and biomarkers to strengthen causal inference and improve translational relevance.

## Data Availability

Publicly available datasets were analyzed in this study. All included datasets are derived from publicly available peer-reviewed studies cited in this article. Full texts of the included studies can be accessed via their respective journal platforms, PubMed, and other academic databases using the DOIs and publication details provided in the reference list.
