# Supplementary material for: Association between abdominal obesity and cognitive outcomes in middle-aged adults: a systematic review
Source: Front Nutr. 2026 Sep 8;13:1879986. doi: 10.3389/fnut.2026.1879986 (PMC13593467; doi:10.3389/fnut.2026.1879986)
Supplement: Supplementary file 1 [file Table_1.docx]

**Supplementary Material**

**Supplementary Table S1. Detailed Search Strategy**

**1. PubMed Search Strategy**

**Database**: PubMed

**Time frame**: January 1, 2000 – April 30, 2026

**Language**: English and Chinese

**Filters**: Humans

#1 "Obesity, Abdominal"[Mesh]

OR "Abdominal obesity"[tiab]

OR "Central obesity"[tiab]

OR "Visceral fat"[tiab]

OR "Waist circumference"[tiab]

OR "Waist-to-hip ratio"[tiab]

OR "Waist-to-height ratio"[tiab]

OR "WHR"[tiab]

OR "WHtR"[tiab]

#2 "Cognition"[Mesh]

OR "Cognitive Dysfunction"[Mesh]

OR "Cognition Disorders"[Mesh]

OR "Cognitive function"[tiab]

OR "Cognitive performance"[tiab]

OR "Cognitive decline"[tiab]

OR "Mild cognitive impairment"[tiab]

OR "MCI"[tiab]

OR "Dementia"[tiab]

OR "Alzheimer*"[tiab]

OR "Memory"[tiab]

OR "Executive function"[tiab]

#3 "Middle Aged"[Mesh]

OR "Middle-aged"[tiab]

OR "Midlife"[tiab]

OR "Adults aged 45–59"[tiab]

#4 #1 AND #2 AND #3

Filters applied: Humans; Publication date from 2000/01/01 to 2026/04/30

**2. Embase Search Strategy**

**Database**: Embase (via Elsevier)

**Time frame**: January 1, 2000 – April 30, 2026

**Language**: English and Chinese

**Filters**: Humans

#1 'abdominal obesity'/exp

OR 'central obesity':ti,ab

OR 'visceral fat':ti,ab

OR 'waist circumference':ti,ab

OR 'waist hip ratio':ti,ab

OR 'waist height ratio':ti,ab

#2 'cognition'/exp

OR 'cognitive function'/exp

OR 'cognitive impairment':ti,ab

OR 'cognitive decline':ti,ab

OR 'mild cognitive impairment':ti,ab

OR 'dementia':ti,ab

OR 'alzheimer disease':ti,ab

OR 'memory':ti,ab

OR 'executive function':ti,ab

#3 'middle aged'/exp

OR 'middle aged' OR 'middle-aged':ti,ab

OR 'midlife':ti,ab

#4 #1 AND #2 AND #3

Limits: Human; 2000–2026

**3. Web of Science Search Strategy**

**Database**: Web of Science Core Collection

**Time frame**: January 1, 2000 – April 30, 2026

**Language**: English

TS = (

("abdominal obesity" OR "central obesity" OR "visceral fat" OR

"waist circumference" OR "waist-to-hip ratio" OR "waist-to-height ratio")

AND

("cognitive function" OR "cognitive decline" OR "cognitive impairment" OR

"dementia" OR "mild cognitive impairment" OR "Alzheimer" OR "memory" OR "executive function")

AND

("middle-aged" OR "midlife")

)

Refined by: Document Types (Article OR Clinical Trial)

Timespan:January 1, 2000 – April 30, 2026

**4. Cochrane Library Search Strategy**

**Database**: Cochrane Library

**Time frame**: January 1, 2012 – April 30, 2026

("abdominal obesity" OR "central obesity"OR "visceral fat"OR "waist circumference" OR "waist-to-hip ratio")

AND

("cognitive function" OR "cognitive decline" OR"cognitive impairment" OR "dementia" OR "MCI")

AND

("middle-aged" OR "midlife")

**5. CNKI（中国知网）检索策略**

**数据库**： CNKI

**时间范围**： January 1, 2000 – April 30, 2026

**语言**： Chinese

主题 = (

（"腹型肥胖" OR "中心性肥胖" OR "内脏脂肪" OR "腰围" OR "腰臀比"OR "腰高比"）

AND

（"认知功能" OR "认知下降" OR "认知障碍"认知衰退" OR"轻度认知功能障碍"OR "痴呆" OR "阿尔茨海默病"）

AND

（"中年" OR "中年人" OR "中年群体"）

)

The search strategies were adapted for each database using a combination of controlled vocabulary (e.g., MeSH and Emtree terms) and free-text keywords. Boolean operators (AND, OR) were used to combine search terms. Reference lists of included studies and relevant reviews were also manually screened to identify additional eligible studies.
